# Statins: benefits and risks revisited

**DOI:** 10.18632/aging.102056

**Published:** 2019-07-14

**Authors:** Miroslav Balaz, Christian Wolfrum

**Affiliations:** 1 Institute of Food, Nutrition and Health, ETH Zürich, Schwerzenbach 8603, Switzerland

**Keywords:** statins, mevalonate pathway, protein prenylation, brown adipose tissue, type 2 diabetes

Prevalence of obesity has been increasing worldwide and has reached pandemic proportions. Obesity, defined as excessive fat accumulation in the body, is associated and deemed causal for many co-morbidities such as atherosclerosis, hepatic steatosis, hyperlipidemia and type 2 diabetes (T2D), to name but a few. These medical complications of obesity have a significant impact on life quality and can reduce life expectancy by up to 14 years [1]. Since obesity is mainly caused by a long-term imbalance between food intake and energy expenditure, lifestyle modifications are in the majority of obese individuals sufficient to improve the metabolic phenotype. Nevertheless, a large number of studies in the last decades were aimed to devise novel therapeutic strategies to increase energy expenditure.

Activation of brown adipose tissue (BAT) is one of the most appealing strategies to reduce our metabolic efficiency, as brown adipocytes have the unique capacity to dissipate energy in the form of heat through a process called adaptive non-shivering thermogenesis. Interest in BAT research substantially increased in the last decade after it was shown that physiologically relevant amount of BAT can be found in adult humans and activated in almost all healthy subjects by a mild cold exposure as well as by administration of a selective β3-adrenergic receptor agonist. So far, sympathetic stimulation is the only mechanism, which was shown to be effective in stimulation of BAT activity in a clinical setting, but unfortunately, the high doses, which are required to activate BAT, might lead to adverse effects on the cardiovascular system, which could prevent their widespread use [2]. Hence, the identification of novel mechanisms driving BAT development and activity has been the focus of recent research.

Recently, we reported that mevalonate pathway, the target of statin action is essential for recruitment of brown/beige adipocytes in mice as well as in humans [3]. We first identified enrichment of the key enzymes providing substrate for mevalonate synthesis in human BAT biopsies and we confirmed increased activity of this pathway in murine BAT, compared to WAT. Inhibition of mevalonate pathway with statins strongly suppressed browning of human white adipocytes *in vitro* and WAT in mice *in vivo.* In a retrospective clinical study, we found an inverse correlation between statin use and presence of active BAT in adult humans and this finding is supported by a small prospective clinical trial, which shows that a short-term fluvastatin therapy is sufficient to suppress thermogenic gene expression in BAT of metabolically healthy young adult men [3]. In addition, we could demonstrate that the adverse statin action on BAT functionality resulted from depletion of isoprenoids, which are synthesized as intermediates in mevalonate pathway and serve as substrates for protein prenylation. This posttranslational modification is essential for proper function of small GTP-binding proteins, which are involved in a large number of cellular processes, including regulation of F-actin formation, adipogenesis and white adipocyte browning. Importantly, stimulation of protein geranylgeranylation promotes white adipocytes browning *in vitro* as well as *in vivo* [3]. Further clinical studies are warranted to substantiate our results and to determine whether protein geranylgeranylation or individual small GTPases might be used for therapeutic strategies to promote thermogenic activity of fat cells and thereby increase whole-body energy expenditure.

**Figure 1:**
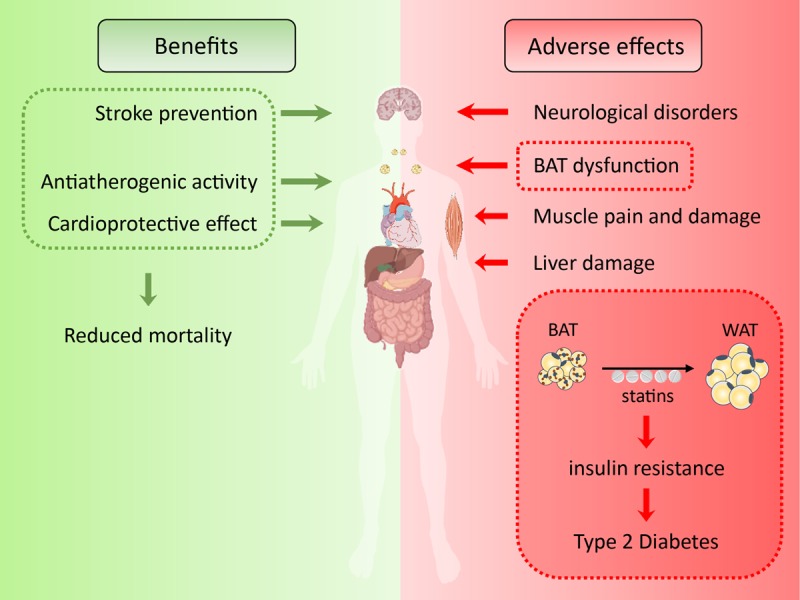
**Statin benefits and adverse effects.** Statin therapy shows cardioprotective and antiatherogenic effect, thereby reducing mortality. However, statins show also adverse effects, particularly on brown adipose tissue (BAT), liver and muscle function. We suggest that BAT dysfunction might be the mediator of statin induced insulin resistance and type 2 diabetes.

Statins are the most widely prescribed and best-selling drugs of all times. They inhibit activity of HMG-CoA reductase, the rate-limiting enzyme of mevalonate pathway, thereby lowering endogenous production and circulating LDL cholesterol levels, which is associated with reduced incidence of cardiovascular disease and mortality. Even though liver is the primary target of statin action, several cholesterol-independent effects in other organs and tissues have been described, including improvement of endothelial function, anti-inflammatory and anti-proliferative properties, as well as stabilization of atherosclerotic plaques [4]. These pleiotropic effects result mostly from inhibition of isoprenoid synthesis. Statins are generally well tolerated; however, several statin trials and large meta-analyses revealed an increased incidence of T2D. Is has been estimated, that treatment of 10,000 patients for 5 years with a standard statin regimen would cause about 50-100 new cases of T2D [5]. A recent study in a population-based Metabolic Syndrome in Men cohort of over 8,700 participants showed an increase in T2D incidence for participants on statin treatment, by 46% after adjustment for confounding factors [6]. The clear link between statin treatment and the increased incidence of T2D has not been mechanistically addressed yet, but seems to be associated primarily with insulin resistance, as statin therapy was associated with a 24% reduction in insulin sensitivity compared with individuals without statin therapy [6]. Furthermore, genetic variants leading to reduced activity of HMG-CoA reductase also confer a higher risk of T2D in the absence of statins, suggesting a decisive role for the target of statins rather than an off-target effect [7]. Furthermore, adipose-specific ablation of HMG-CoA reductase significantly impaired glucose and insulin tolerance in mice [8], indicating that the diabetogenic effect of statins might be due to inhibition of mevalonate pathway in adipose tissue. Especially BAT is an important regulator of systemic glucose and lipid homeostasis. It acts as a sink for calories, thereby protecting muscle, liver and pancreas from excessive lipid deposition and insulin resistance. This is in line with our findings that the inhibition of mevalonate synthesis adversely affects brown adipocyte signalling [3] and might thus explain, at least in part, the increased incidence of T2D as a consequence of reduced BAT activity (Figure 1). However, further clinical studies with different statins, dosages and treatment duration are needed to unveil the exact mechanism of detrimental statin action on the whole-body metabolism.
